# Recommendations from the *Sociedade Portuguesa de Cuidados Intensivos* and Infection & Sepsis Group for intensive care approach to COVID-19

**DOI:** 10.5935/0103-507X.20200002

**Published:** 2020

**Authors:** João João Mendes, Paulo Mergulhão, Filipe Froes, José Artur Paiva, João Gouveia

**Affiliations:** 1 Sociedade Portuguesa de Cuidados Intensivos - Lisboa, Portugal.; 2 Intensive Medicine Service, Hospital Prof. Doutor Fernando da Fonseca EPE - Lisboa, Portugal.; 3 Polyvalent Intensive Care Unit, Hospital CUF Infante Santo - Lisboa, Portugal.; 4 Microbiology Institute, Faculdade de Medicina, Universidade de Lisboa - Lisboa, Portugal.; 5 Infection and Sepsis Group - Lisboa, Portugal.; 6 Polyvalent Intensive Care Unit, Hospital Lusíadas Porto - Porto, Portugal.; 7 Medical-Surgical Intensive Care Unit, Hospital Pulido Valente, Centro Hospitalar Universitário de Lisboa Norte EPE - Lisboa, Portugal.; 8 College of Intensive Care Specialties, Ordem dos Médicos - Lisboa, Portugal.; 9 Faculdade de Medicina, Universidade do Porto - Porto, Portugal.; 10 Intensive Medicine Service, Centro Hospitalar Universitário de Lisboa Norte EPE - Lisboa, Portugal.

**Keywords:** Coronavirus infections, COVID-19, SARS virus, Sepsis, Intensive care units, Infecções por coronavirus, COVID-19, Virus da SARS, Sepse, Unidades de terapia intensiva

## Abstract

Current COVID-19 epidemics was declared on December 31, 2019 at the Wuhan city seafood market, rapidly spreading throughout China, and later reaching several countries (mainly South Korea, Japan, Italy and Iran) and, since March 1, reaching Portugal. Most of the infected patients present with mild symptoms, not requiring hospitalization. Among those admitted to the hospital, 6% to 10% require admission to the intensive care unit. These recommendations are aimed to support the organization of intensive care services to respond COVID-19, providing optimized care to the patient and protection for healthcare professionals.

## Abstract (pt)

A atual epidemia de COVID-19 foi declarada em 31 de dezembro de 2019 no mercado de frutos do mar da cidade de Wuhan, com rápida disseminação na China e, posteriormente, envolvendo múltiplos países (como maior expressão na Coreia do Sul, Japão, Itália e Irã) incluindo, desde 1º de março, Portugal. A maioria dos doentes infetados apresenta doença ligeira sem necessidade de hospitalização. Dentre os internados, de 6% a 10% necessitam de cuidados intensivos. As presentes recomendações visam facilitar a organização dos serviços de medicina intensiva para a resposta ao COVID-19, proporcionado os melhores cuidados aos doentes e protegendo os profissionais de saúde.

## INTRODUCTION

COVID-19, short for coronavirus disease 2019, is a clinical disease caused by SARS-CoV-2, a simple positive-sense RNA genome virus, belonging to the coronaviruses (CoV) family. There are four strains (HCoV-229E, HCoV-NL63, HCoV-OC43, and HCoV-HKU1) with seasonal circulation among human populations, most frequently causing mild-severity respiratory infections (such as common cold) and, rarely, viral pneumonia.^(1)^ Before the emergence of SARS-CoV-2,^(2)^ two strains causing epidemic outbreaks from zoonotic origin were described: SARS-CoV-1, causing severe acute respiratory syndrome (SARS) originated from bats and transmitted to the African civet, and later to humans in 2002; and MERS-CoV, causing the Middle East respiratory syndrome (MERS) originated from bats, transmitted to camelids, and later, to humans in 2012.^(1)^

On December 31, 2019, a pneumonia cluster from an unknown cause was identified in the Chinese city of Wuhan (Hubei province), later associated with the Huanan Seafood Wholesale Market. The respiratory samples from these patients lead to the identification of a new virus (SARS-CoV-2) with an epidemic dissemination throughout China and progressively spreading to multiple countries (mostly South Korea, Japan, Italy, and Iran),^(3)^ and since March 2, to Portugal.^(4)^ Its contagion capacity is due to a significant attack rate (R⌀, number of new cases generated from one single confirmed case) that has been estimated to be from 2.5 to 2.9, although potentially affected by public health interventions.^(5)^ High risk of nosocomial transmission has been observed, as well as contamination of healthcare professionals.^(6)^

The median incubation time is four days, but it may last until 14 days,^(6)^ and the SARS-CoV-2, similar to SARS-CoV-1, links to angiotensin 2 converting enzyme receptors located on type II alveolar cells, causing diffuse alveolar and direct cytopathic injury.^(7)^ Most of the infected patients (> 80%) present with mild disease, with no need for admission to the hospital. Among those admitted to the hospital, 6% to 10% require referral to intensive care.^(3,6)^

These recommendations are aimed to support the organization of intensive care services to respond to COVID-19, providing optimized care for patients and protection of healthcare professionals.

## 1. CLINICAL SYNDROMES ASSOCIATED WITH COVID-19

### Recommendation 1

It is recommended to adapt the World Health Organization (WHO) criteria for definition of clinical syndromes associated with COVID-19 (Attachment 1).

## 2. CRITERIA FOR ADMISSION TO INTENSIVE CARE SERVICES

### Recommendation 1

It is recommended that patients with criteria for severe pneumonia (Attachment 1) are early referred to intensive care services, in order to discuss the decision and timing for referring to the intensive care service.

### Recommendation 2

It is recommended that admission to intensive care is not based on strict criteria, but on case-by-case assessment, each time including perception of severity by the clinician and global management of the available resources.

### Recommendation 3

It is recommended that, in case of extreme lack of resources, explicit priority admission criteria to intensive care are in place.

## 3. ISOLATION AND INDIVIDUAL PROTECTION EQUIPMENT

### Recommendation 1

Isolation in individual rooms with negative pressure and gates, privative toilet and ventilation system with capacity for 6 - 12 air changes per hour. Once exhausted these resources, patients are recommended to be isolated in individual rooms with a ventilation system with capacity for 6 - 12 air changes per hour. When no isolation individual rooms are available, cohort isolation is recommended, keeping a minimum distance of 1 meter between patients’ units.

### Recommendation 2

Visits restriction is recommended for all patients and limiting the number of professionals in contact with the patient (ideally, dedicated professionals), implementing alternative remote ways for communication between patient and family, and the clinical team, patient and family, independent of the isolation site.

### Recommendation 3

All healthcare professionals involved in clinical care are recommended to use universal precautions, contact precautions, and droplet precautions. These include wearing specific individual protection equipment, disposable (one use) and impermeable coat, surgical mask, eye protection, and clean gloves. During potentially generating aerosols care (e.g. intubation, aspiration of secretions and bronchoscopy), or prolonged contact (> 15 minutes), and/or close contact (e.g. placing central venous catheter, surgery, cardiorespiratory reanimation maneuvers), airway precautions are recommended. These include wearing specific individual protection equipment, disposable (one use) and impermeable: coat (with long sleeve and elastic cuff, and covering until the mid of legs or ankles), cap, FFP2/FFP3 mask (appropriately fit to the face), eye protection (with lateral protection), gloves (reaching above the coat sleeves), and shoe protection (ideally impermeable shoes for exclusive use on the isolation area or impermeable shoe protection). It is recommended that the integral protection outfit (impermeable, including hat and neck protection) is limited to trained professionals with practical experience in their use. Once the use of nebulizers, noninvasive mechanic ventilation, and high-flow nasal cannulas are potential aerosol generators, airway protection measures are recommended to be equally used during the clinical care of these patients.

### Recommendation 4

Order and technic for putting on and removing individual protection equipment should be strictly adhered to (ideally using a mirror or observation by another healthcare professional). Particularly during removal, it is recommended additional care to prevent self-contamination and contamination other people and the environment.

### Recommendation 5

It is recommended that all healthcare professionals are trained and have practical experience putting on and removing individual protection equipment before any contact with patients.

### Rational

We followed the recommendations from the Portuguese General Health Management^(8)^ based on WHO^(9)^ and European Centre for Disease Prevention and Control (ECDC)^(10)^ on strategies for prevent and control SARS-CoV-2 infection mainly on the prevention of transmission in healthcare facilities, based on recommendations previously issued for MERSCoV1 and SARS-CoV1.

## 4. HOSPITAL ORGANIZATION

### Recommendation 1

It is recommended the Intensive Care Service management to manage all hospital level 2 (intermediate) and level 3 (intensive) beds, (independently of the service they are located) in close cooperation with the Clinical Director, General Health Management and the Ministry of Health.

### Recommendation 2

It is recommended that, in hospitals with more than one intensive care unit, a cohort area is set for confirmed COVID-19 critical patients, and to consider setting a cohort area for suspected critical patients (for temporary stay), and criteria for its activation are established.

## 5. INFECTION DIAGNOSIS

### Recommendation 1

It is recommended that the microbiologic diagnosis use a real-time polymerase chain reaction test (real-time PCR) to analyze samples from upper airways (swabs of nasopharynx and oropharynx exudates), whenever possible associated with a lower respiratory tract sample (bronchial secretions collected from endotracheal aspirate).

### Recommendation 2

It is not recommended to perform bronchoscopy exclusively to collect samples from the lower respiratory tract.

### Recommendation 3

Whenever there is high clinical suspicion (especially if a computed tomography scan shows disease evidence) and microbiologic testing is negative, it is recommended to repeat the test to either confirm or rollout infection, preferably with samples from the lower respiratory tract.

### Recommendation 4

Blood culture samples are recommended (at least two blood culture sets, aerobic and anaerobic) and a sample from the lower respiratory tract to search for other microbiologic agents.

### Rational

Real-time PCR test (RT-PCR) is a highly specific test to identify the presence of SARS-CoV-2, and patients with greater viral load (most frequently during the disease) may be more likely to show a positive test. However, for patients with suspected COVID-19 and a negative RT-PCR test, repeating the test was positive in 23% of the cases (conversion over time), pointing to a sensitivity lower than 80%. In these cases, whenever possible a chest computed tomography scan should be performed, searching for disease evidence before real-time PCR.^(11,12)^ Coinfection with other microbiological agents, especially in the event of a septic shock, is frequent.^(11^

## 6. INITIAL THERAPY FOR SUSPECTED/CONFIRMED CASES

### Recommendation 1

All severe pneumonia patients are recommended to receive oxygen therapy, started with 4L/minute nasal cannula and titrated to oxygen saturation (SpO_2_) ≥ 92%, without humidification.

### Recommendation 2

A conservative fluid therapy strategy is recommended, especially in the absence of shock.

### Rational

We adhered to WHO recommendations,^(13)^ highlighting that no humidification is required for oxygen flows < 4L/minute^(14)^ and that the use of bubble humidifiers with oxygen flows ≥ 5L/minute produces aerosols that increase the risk of transmitting microorganisms.^(15)^ The initial presentation of COVID-19 is rarely of a septic shock, and the most frequent cause of death is hypoxemic respiratory failure,^(16)^ aggravated by inappropriate fluid therapy.^(17^

## 7. INDICATIONS AND STRATEGY FOR HIGH-FLOW NASAL CANNULA OXYGEN THERAPY

### Recommendation 1

It is recommended not to use high-flow nasal cannula oxygen therapy in patients during the active viral replication phase.

### Recommendation 2

It is recommended that, if a decision to start a high-flow nasal cannula oxygen therapy is made:

(1) that the technique is started at the intensive care service in a highly monitored environment, allowing to prevent delaying intubation in cases of failure to respond;
(2) that low flows are preferred (15 - 30L/minute), with the patient wearing a surgical mask (whenever possible); and
(3) that healthcare professionals use contact precautions for droplets and airway (ideally in negative pressure rooms).

### Rational

Nasal cannula high-flow oxygen therapy has been successfully used in hypoxemic respiratory failure patients,^(18)^ however it can potentially delay endotracheal intubation^(19)^ and increase the risk of aerosols generation.^(20^

## 8. NONINVASIVE VENTILATION: INDICATIONS AND STRATEGY

### Recommendation 1

In these patients it is recommended not to use noninvasive ventilation, especially during the viral active replication phase.

### Recommendation 2

It is recommended that, if a decision to start noninvasive ventilation is made:

(1) it is started at the intensive care service under intensive monitorization, preventing delays of endotracheal intubation in case of failure to respond;
(2) to use maximal sealing masks or helmets, as well double-circuit ventilators (e.g. with an expiratory loop) with a high-efficiency application; and
(3) the healthcare professionals use contact precautions, both for droplets and airways (ideally in negative pressure rooms).

### Rational

Results of noninvasive ventilation are worse than conventional oxygen therapy, and high-flow nasal cannula oxygen therapy in patients with hypoxemic respiratory failure,^(18)^ and was associated with increased therapeutic failure in patients with MERS,^(21)^ as well as it leads to an increased risk of generating aerosols.^(20,22^

## 9. INDICATIONS FOR OROTRACHEAL INTUBATION

### Recommendation 1

**Figure 1:**
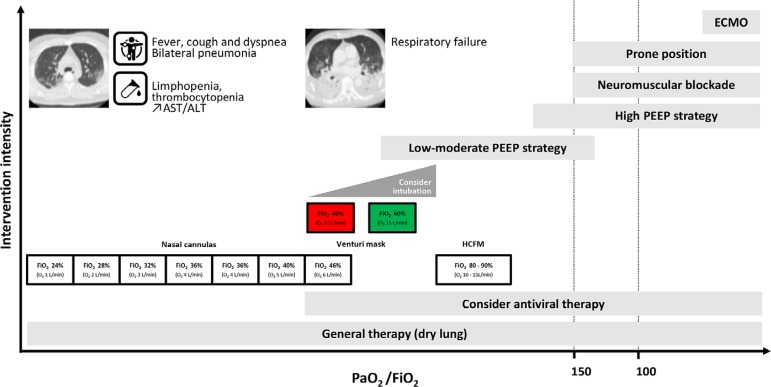
Therapeutic options (support and directed) for COVID-19. AST - aspartate aminotransferase; ALT - alanine aminotransferase; FiO2 - fraction of inspired oxygen; PEEP - positive end-expiratory pressure; HCFM - high-concentration face mask; ECMO - extracorporeal membrane oxygenation.

An early intubation strategy is recommended as opposed to a late intubation strategy (Figure 1).

### Rational

In a context of hypoxemic respiratory failure, a delay in endotracheal intubation is associated with increased mortality^(23)^ as previously observed in COVID-19 patients.^(20)^

## 10. OROTRACHEAL INTUBATION STRATEGY

### Recommendation 1

It is recommended that endotracheal intubation is performed by an experienced professional (who is more likely to succeed in a first attempt), wearing contact precautions for droplets and airway (ideally in a negative pressure room).

### Recommendation 2

It is recommended that endotracheal intubation is performed using:

(1) pre-oxygenation with a high-concentration facial mask or a Mapleson C type balloon, connected to a high-efficiency respiratory filter, always avoiding manual inflations;
(2) rapid sequence intubation technique;
(3) video laryngoscopy using a disposable blade;
(4) after intubation, clamping the tube until connection to a manual ventilator (or a Mapleson C type balloon) or to the mechanical ventilator adapted to a high efficiency filter;
(5) confirm the intubation by capnography/capnometry followed by chest X-ray (no auscultation).

### Rational

Endotracheal intubation is associated with a clear risk of generating aerosols,^(20,22)^ and all strategies should be in place to minimize the risk of transmission to the healthcare professionals.^(24^

## 11. INDICATIONS AND STRATEGY FOR INVASIVE MECHANIC VENTILATION AND THERAPEUTIC ADJUVANTS

### Recommendation 1

A classic ventilation strategy is recommended, based on the Acute Respiratory Distress Syndrome Network protocol (tidal volume of 6mL/kg ideal body weight, with an upper limit for plateau pressures < 30cmH2O), using a table for high positive end-expiratory pressure (PEEP) in patients with moderate to severe acute respiratory distress syndrome (ARDS), associated with a driving pressure < 15cmH2O and a respiratory rate to maintain pH > 7.25.

### Recommendation 2

It is recommended early prone position ventilation in patients with partial oxygen pressure/fraction of inspired oxygen (PaO2/FiO2) < 150mmHg, for minimum 16 hours periods.

### Recommendation 3

Neuromuscular blockers are recommended to be used for ≤ 48 hours in patients with PaO2/FiO2 < 150mmHg.

### Recommendation 4

In patients with active viral replication, it is recommended that spontaneous breathing test, when indicated, is performed with support pressure using a closed circuit, instead of a T tube.

### Rational

We adhere to the WHO^(13)^ recommendations, highlighting the strategies shown effective in ARDS.^(25-28^

## 12. INDICATIONS FOR EXTRACORPOREAL MEMBRANE OXYGENATION

### Recommendation 1

It is recommended adherence to current criteria for referral to extracorporeal membrane oxygenation (ECMO), based on a case-by-case assessment, including the perception of severity by the clinician and overall resources management.

### Recommendation 2

In case of an extraordinary scarcity of resources, it is recommended to explicit the criteria for referral to ECMO.

### Rational

ECMO is therapy to be considered in a context of refractory hypoxemic respiratory failure,^(29)^ but, although already in use, its benefit in COVID-19 is still unclear.^(30^

## 13. INDICATIONS FOR BRONCHIAL FIBROSCOPY

### Recommendation 1

It is recommended not to perform bronchial fibroscopy, except for well-established indications.

### Recommendation 2

It is recommended that, if a decision for a bronchial fibroscopy is made, it should be performed by the most experienced professional wearing airway precautions (in negative pressure rooms).

### Rational

Bronchial fibroscopy is associated with a clear risk of generating aerosols^(22)^, and all strategies should be in place to minimize the risk of transmission to healthcare professionals.^(11^

## 14. INHALATION THERAPY

### Recommendation 1

When clinically indicated, it is recommended inhalation therapy not to be performed using pneumatic, ultrasonic or oscillatory membrane devices.

### Rational

Inhalation therapy using pneumatic, ultrasonic or oscillatory membrane devices is associated with a clear risk of generating aerosols^(22)^, and all strategies should be in place to minimize the risk of transmission to healthcare professionals.^(11^

## 15. INDICATION FOR CORTICOSTEROID THERAPY

### Recommendation 1

It is recommended to avoid the administration of corticosteroids, unless if indicated for other reasons (e.g. chronic obstructive pulmonary disease exacerbation or septic shock, according to the Surviving Sepsis Campaign guidelines).

### Rational

Current evidence point to a lack of corticosteroids benefit on mortality, causing delayed viral clearance in patients infected with SARS-CoV-1 e MERS-CoV.^(31,32^

## 16. INDICATIONS AND STRATEGY FOR ANTIBIOTIC THERAPY

### Recommendation 1

In case of severe pneumonia (while waiting for SARS-CoV-2 identification) in times of seasonal influenza, in association with antibiotic therapy to start to start influenza therapy (Table 1), to be reassessed after laboratory tests and culture are available.

**Table 1:** Antibiotic therapy schedule for severe pneumonia (alternatives should be weighted according to modifying factors)

[Ceftriaxone 2g/day (or 1g every 12 hours) intravenously or amoxicillin/clavulanic acid 2.2g every 8 hour intravenously
+ Azithromycin 500mg/day intravenously, or clarithromycin 500mg every 12 hours intravenously] *
+ Oseltamivir 75mg (tablet), 2 tablets (150mg) every 12 horas, enteral

* Levofloxacin 500mg/day intravenously (if intolerance/allergy to first-line agents).

### Recommendation 2

It is recommended that in case of severe pneumonia (with identified SARS-CoV-2) with a septic shock, to start antibiotic therapy until culture results are available, allowing to confirm or rule out coexisting bacterial infection.

### Recommendation 3

It is recommended that, in case of severe pneumonia (with identified SARS-CoV-2), in the absence of a septic shock, starting antibiotic therapy to be decided on a case-by-case basis and reconsidered in case of negative results.

### Rational

Coinfection with other microbiological agents, especially in case of a septic shock, is frequent.^(11)^ Mortality associated with septic shock is high and associated with a delayed start of effective antibiotic therapy.^(33^

## 17. INDICATIONS AND STRATEGY OF ANTIVIRAL THERAPY

### Recommendation 1

There is no evidence from randomized controlled trials to recommend any specific antiviral therapy for COVID-19 patients.

### Recommendation 2

Experimental therapy (Table 2) may be considered for patients with clinical criteria for severity.

**Table 2:** Antiviral schedule for critical patients

Remdesivir 200mg intravenously (loading dose, day 1) followed by remdesivir 100mg/day intravenously (maintenance dose, days 2 to 10)*
+
Chloroquine phosphate 250 mg (tablet), 2 tablets (500 mg) every 12 hours, enteral† for 5 to 20 days (as determined by clinical progress)

* Se/enquanto remdesivir não disponível: lopinavir/ritonavir 200/50mg (comprimido), 2 comprimidos (400/100mg) a cada 12 horas, via entérica OU lopinavir/ritonavir 80/20mg/mL (solução oral), 5mL (400/100mg) a cada 12 horas, por via entérica;

† ou, em alternativa, hidroxicloroquina 200mg (comprimido), 1 comprimido 2 vezes/dia, por via entérica.

### Rational

No antiviral therapy was proven effective for COVID-19. Several randomized controlled trials are ongoing.^(34)^ Limited evidence is available for the use of lopinavir/ritonavir,^(35,36)^ chloroquine^(37)^ and remdesivir,^(38,39)^ that were included in the Italian therapy protocol.^(40)^ Retrospective data on SARS-CoV point to higher efficacy of early started therapy (< 48 hours),^(35)^ however efficacy after this time is not ruled out, consistent with data for influenza virus infection.

## Attachment 1 - Clinical Syndromes Associated with COVID-19

**Table:**

Uncomplicated disease	Upper respiratory symptoms (e.g. odynophagia, cough, and nasal congestion) either or not associated with fever and myalgias, with no criteria for severity.
Non-severe pneumonia	Fever associated with respiratory symptoms and a radiologic infiltrate, without criteria for severe pneumonia.
Severe pneumonia	According to the IDSA/ATS^(41)^ criteria
	- ≥ 1 major criterion
	Septic shock
	Mechanic ventilation
	- ≥ 3 minor criteria
	Confusion
	Respiratory rate ≥ 30rpm
	Hypotension with aggressive fluid resuscitation
	Hypothermia (central temperature < 36ºC)
	PaO_2_/FiO_2_ ≤ 250mmHg
	BUN ≥ 43mg/dL
	Leucopenia (leucocytes ≤ 4,000/*µ*L)
	Thrombocytopenia (platelets ≤ 100,000/*µ*L)
SDRA
	Acute hypoxemic respiratory failure (PaO_2_/FiO_2_ ≤ 300 with PEEP/CPAP ≥ 5cmH_2_O) (starting < 1 week after know risk factor) characterized by bilateral opacities (not explained by effusion, atelectasis or nodules) and not explained by heart failure or fluid overload (exclusion by means of clinical, laboratory and echocardiographic assessment).
Sepse	According to the Surviving Sepsis Campaign^43^ criteria
	Life-threatening organs dysfunction (clinically evidenced as acute ≥ 2 points increase of SOFA score) caused by dysregulated host response to infection
Choque séptico	According to the Surviving Sepsis Campaign^43^ criteria
	A subset of patients with sepsis (clinically identified for serum lactate > 2 mmol/L in the absence of hypovolemia and requiring vasopressors to maintain mean blood pressure ≥ 65 mmHg), with unique circulatory/cellular/metabolic changes entailing increased mortality.

Source: adapted from World Health Organization (WHO). Clinical management of severe acute respiratory infection when Novel coronavirus (2019-nCoV) infection is suspected. Interim guidance. 28 January 2020. Available from: https://www.who.int/docs/default-source/coronaviruse/clinical-management-of-novel-cov.pdf.^(13)^

IDS/ATS - Infectious Diseases Society of America/American Thoracic Society; PaO_2_/FiO_2_ - partial oxygen pressure/fraction of inspired oxygen; PEEP/CPAP - positive end-expiratory pressure/continuous positive airway pressure; ARDS - adult respiratory distress syndrome; SOFA - Sequential Organ Failure Assessment.
